# Bone loss and the aromatase inhibitors

**DOI:** 10.1038/sj.bjc.6602691

**Published:** 2005-08-15

**Authors:** J Lester, R Coleman

**Affiliations:** 1 Academic Unit of Clinical Oncology, Cancer Research Centre, Weston Park Hospital, Sheffield S10 2SJ, UK

**Keywords:** aromatase inhibitors, bone loss, tamoxifen, steroidal, nonsteroidal

## Abstract

The increasing use of systemic adjuvant therapies has considerably improved the prognosis from early breast cancer. However, some of these therapies affect bone metabolism, resulting in osteoporosis. Aromatase inhibitors lower circulating oestrogen levels to almost unrecordable levels in postmenopausal women, predisposing them to bone loss with an increase in fracture risk. Ongoing clinical trials are favouring the use of the aromatase inhibitors over tamoxifen and this may advocate greater use of these drugs in the future. Strategies for the identification and management of treatment-induced bone loss are currently being defined.

The incidence of breast cancer is rising steadily and at present is the most common cancer in women. New therapeutic options are improving survival and this is exposing an increasing population to the late complications of breast cancer and its treatment. In the last few years, research has shown that breast cancer has important implications for bone health. Of major concern is the development of bone metastasis, but many breast cancer patients are at increased risk for developing osteoporosis, which is also associated with marked morbidity and mortality (Adachi *et al*, 2002; Cummings and Melton, 2002; Hasserius *et al*, 2003; Marks *et al*, 2003).

The pathogenesis of osteoporosis is complicated with many factors involved, although oestrogen deficiency is perhaps the most important factor. The increasing use of chemotherapy in young women leading to premature menopause, and the introduction of aromatase inhibitors into the adjuvant setting for postmenopausal women have improved outcome, but have clinically important consequences on bone mineral density (BMD) and fracture risk.

Bone mineral density is a surrogate marker of lifetime exposure to oestrogen and therefore is a risk factor for developing breast cancer (Cauley *et al*, 1996; Zhang *et al*, 1997). Consequently, newly diagnosed breast cancer patients have a higher average BMD than the normal age-matched population (van der Klift *et al*, 2003). Despite this, it is becoming increasingly evident that breast cancer and its treatment are associated with the development of osteoporotic fracture. In a study by Kanis *et al* (1999), 1210 patients with breast cancer but no evidence of bone metastasis were investigated. The incidence of vertebral fracture was 5.4% in the newly diagnosed breast cancer patients compared with only 1.5% in age-matched women without breast cancer.

The reductions in circulating oestrogen levels that occur at the menopause are associated with a rapid deterioration in bone mass by as much as 3% per year for the first 5 years after the menopause (Riggs *et al*, 1998). After the menopause, intrinsic production of oestrogen from androgens largely occurs in nonovarian tissues such as fat, muscle, skin and liver as a result of the activity of the aromatase enzyme complex (Sluijmer *et al*, 1995). The resulting low levels of circulating oestrogen help to prevent a more exaggerated bone loss (Cummings *et al*, 1998a).

## CAUSES OF ACCELERATED BONE LOSS IN BREAST CANCER

There are many treatments for breast cancer that increase the risk of osteoporosis; these are outlined below (Table 1).

### Chemotherapy

#### Indirect effects

The use of chemotherapy in premenopausal patients commonly induces a sudden deterioration in oestrogen production and often an early menopause. A study by Shapiro *et al* (2001) investigated 49 premenopausal stage 1 and 2 breast cancer patients treated with adjuvant chemotherapy. After 1 year, 35 (71%) patients were found to have ovarian failure and these patients lost on average 4.0% (*P*=0.0001) of the lumber spine BMD after just 6 months. This rate of bone loss was maintained at 12 months with a further 3.7% (*P*=0.0001) of bone loss.

#### Direct effects

Studies on rat skeletons after the administration of methotrexate suggest that chemotherapy may have a direct impact on bone by inhibiting proliferation. After treatment, bone histomorphometry showed a significant reduction in bone volume, strength and mineralisation surface compared with controls (*P*<0.05) (Wheeler *et al*, 1995).

A study by Greep *et al* (2003) assessed postmenopausal patients who are not susceptible to the ovarian suppression caused by chemotherapy. The changes observed in BMD suggested a possible direct effect of chemotherapy. Although no significant change in BMD T-score (based on peak bone mass) was seen, the Z-score (age-adjusted difference from the mean) did fall more rapidly than in control patients. The average change in Z-score for chemotherapy patients was −0.65 (*P*=0.0002) at the total hip and −0.60 (*P*=0.05) at the lumbar spine.

These studies suggest that chemotherapy may have direct effects on bone metabolism, particularly bone formation, but overall, premature ovarian failure is the major cause of chemotherapy-induced bone loss.

### Tamoxifen

Tamoxifen has a complicated mechanism of action with potent antioestrogenic effects on the breast, but also partial oestrogen agonist effects on bone, uterus and lipids. The partial agonist effect appears to have varying impacts on bone metabolism depending on the intrinsic circulating levels of oestrogen. Postmenopausal women receiving tamoxifen therefore may be protected from the development of osteoporosis, due to the mild oestrogen agonist action. Love *et al* (1992) conducted a placebo-controlled trial investigating 140 postmenopausal women with breast cancer receiving tamoxifen. After 2 years, BMD of the lumbar spine increased by 0.61% per year in the tamoxifen group (*P*=0.04) and declined by 1.0% per year in the placebo group (*P*<0.001). A similar study by Kristensen *et al* (1994) also showed that postmenopausal women on tamoxifen therapy had a significantly higher BMD than placebo-treated patients (*P*<0.0074).

**Figure 1:**
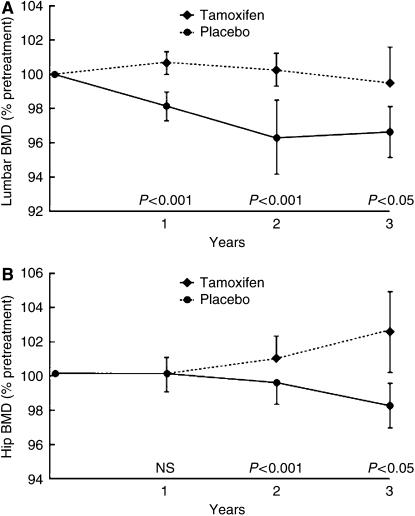
Changes in BMD of (**A**) the hip and (**B**) the spine in premenopausal women on tamoxifen or placebo. Reproduced with permission from Powles *et al* (1996).

Premenopausal women, however, are rendered relatively oestrogen deficient by tamoxifen and as a result may be more prone to osteoporosis in later life. A study by Powles *et al* (1996) investigated 125 premenopausal women and found that they lost on average 1.44% of their lumbar spine BMD every year on tamoxifen treatment. Placebo-treated patients, however, showed a modest gain in their BMD (*P*<0.001) (Figure 1).

### Ovarian ablation/suppression

#### Surgical

Surgical removal of the ovaries is an effective therapy for premenopausal patients with breast cancer (Ingle *et al*, 1986; Conte *et al*, 1989; Taylor *et al*, 1998). It is also clear that the procedure is associated with a rapid decline in oestrogen and bone strength. Hashimoto *et al* (1995) investigated 244 women who were having regular menstrual cycles up until the point of surgical oophorectomy. After 1 year, the mean BMD declined by 10.7%.

#### Drug induced

The gonadorelin analogue, goserelin, is licensed for the treatment of premenopausal patients with advanced breast cancer and is increasingly used in the adjuvant setting. Goserelin induces ovarian failure followed by a rapid decline in circulating oestrogen. As a consequence, BMD can deteriorate by as much as 4.8% within the lumbar spine after just 6 months (Leather *et al*, 1993).

### Aromatase inhibitors

Aromatase inhibitors are potent inhibitors of oestrogen production and at present are commonly used in patients with metastatic disease and in some early breast cancer patients who are unsuitable for treatment with tamoxifen. Two types of aromatase inhibitor are currently available, which have different mechanisms of action. The nonsteroidal agents (anastrozole, letrozole and aminoglutethimide) are reversible inhibitors, while steroidal agents (exemestane and formestane) are irreversible inactivators of the aromatase enzyme. The third-generation aromatase inhibitors anastrozole, letrozole and exemestane are the most powerful drugs available resulting in approximately 96–99% enzyme inhibition (Dowsett *et al*, 1995; Geisler *et al*, 1996, 1998). This marked reduction in oestradiol would be expected to have profound effects on bone physiology. The decision on which drugs to use in the future will depend upon the clarity of the results of trials in metastatic disease and adjuvant therapy, as well as ‘head to head’ comparison of the agents (Cummings, 2002).

#### Anastrozole

Anastrozole has shown clear advantages over megestrol acetate as second-line therapy for advanced breast cancer and to be at least as good as tamoxifen for first-line therapy (Buzdar *et al*, 1998; Nabholtz *et al*, 2000). It is also being used increasingly in the adjuvant setting. Preliminary results from the Arimidex, Tamoxifen Alone or in Combination (ATAC) trial have been published (ATAC, 2002). The study included over 9000 postmenopausal patients who had completed primary therapy for breast cancer and who were eligible for endocrine therapy. Patients were randomised equally between anastrozole, tamoxifen or a combination of both. Results published after a median follow-up of 33.3 months found that anastrozole improved disease-free survival, time to disease recurrence and incidence of contralateral primary breast cancer compared with both tamoxifen and the combination of drugs. Updated results confirmed these early observations (Baum *et al*, 2003).

From the perspective of adverse events, anastrozole was better tolerated except for the occurrence of musculoskeletal side effects and the incidence of fractures, mainly of the spine and wrist. After a median follow-up of 37 months, the incidence of all fractures was 7.1% in the anastrozole group and 4.4% in the tamoxifen group (*P*<0.0001) (ATAC, 2002) (Table 2). Although the fracture rate in anastrozole-treated women appeared to plateau after around 18 months, with no progressive increase in fracture risk (Table 3), the increased fracture risk seen in the anastrozole patients still remained significant (*P*<0.0001) (Howell, 2003; Locker and Eastell, 2003). The fracture rate per 1000 women years was 21.55 with anastrozole compared with 13.44 with tamoxifen. A population of healthy women of similar median age would be expected to have a fracture rate per 1000 women years of 19.10 (Writing Group for the Women's Health Initiative Investigators, 2002).

The bone subprotocol of the ATAC trial investigated 308 patients and assessed them for any changes in BMD and bone turnover markers (Eastell *et al*, 2002). After 2 years treatment with anastrozole, the BMD had fallen on average by 4.0% in the lumbar spine and 3.2% in the hip. There was also a 15% increase in the bone resorption marker N-terminal telopeptide (NTX) and a 21% increase in bone alkaline phosphatase, a bone formation marker. At this early stage, this is clear evidence that anastrozole reduces BMD in postmenopausal women and does not have the bone protective effects of tamoxifen.

#### Letrozole

Letrozole is superior to megestrol acetate in the treatment of advanced breast cancer and is clearly superior to tamoxifen as first-line therapy in high-risk or advanced disease (Buzdar *et al*, 2001; Ellis *et al*, 2001; Mouridsen *et al*, 2001). Recently, Goss *et al* (2003) investigated the role of letrozole after treatment with 5 years of adjuvant tamoxifen. Patients with breast cancer (*n*=5187) were randomised to further treatment with letrozole or placebo after the completion of 5 years of tamoxifen therapy. After a median follow-up of just 2.4 years, the estimated 4-year disease-free survival was 93% in the letrozole group and 87% in the placebo group (*P*<0.001). Only 61 (2.4%) patients treated with letrozole developed recurrent disease compared to 106 (4.1%) in the placebo group. Unfortunately, as the study was discontinued early, the long-term effects of letrozole are not yet known. However, at this early stage, more diagnoses of osteoporosis have been made in the letrozole group compared with the placebo group, at 5.8 and 4.5% respectively (*P*=0.07), suggesting that, like anastrozole, letrozole increases bone loss and fracture risk (Goss *et al*, 2003). The first results of a specific bone subprotocol evaluating changes in BMD and bone markers were presented by Perez *et al* (2004). Letrozole patients (*n*=122) experienced a significant decrease in total hip BMD compared with placebo (*n*=104) from baseline (−3.6 *vs* −0.71%, *P*=0.044) and a significant decrease in lumbar spine BMD (−0.35 *vs* −0.7%, *P*=0.008) at 24 months. Letrozole also increased the bone resorption marker NTX. In addition, more women receiving letrozole became osteoporotic at the L2–4 spine by BMD criteria than placebo-treated women, although the difference was not significant (3.3 *vs* 0%).

Small short-term studies have shown that letrozole has an impact on markers of bone turnover. Harper-Wynne *et al* (2001) found that C-terminal telopeptide (CTX), a marker of bone resorption, increased from a mean of 2300 to 2828 after 3 months of letrozole therapy (*P*<0.005). Over a 12-week period, Goss *et al* (2002) found that letrozole therapy reduced the bone formation marker, bone-specific alkaline phosphatase (BAP), by 20.1% while the bone resorption marker CTX increased by 11.4%. The study also investigated the impact of exemestane on bone turnover as discussed below.

The ZOFAST study is presently recruiting postmenopausal breast cancer patients with normal bone density. Patients are treated with letrozole and randomised to either immediate intravenous zoledronate or to a delayed phase of treatment based on changes in BMD. Subsequent DXA scans will determine if zoledronate can prevent bone loss in these patients and when they should be considered for treatment with a bisphosphonate. Initial results from the study were reported at the end of 2004, with 587 patients having baseline BMD data (Brufsky *et al*, 2004). At that point, there was a 3.33% difference in lumbar spine BMD in favour of upfront treatment with zoledronate and a 2.42% difference in favour of upfront treatment with total hip BMD.

#### Exemestane

Exemestane is a steroidal aromatase inhibitor, which is also superior to megestrol acetate (Kaufmann *et al*, 2000). There are recent data indicating superior efficacy to tamoxifen (Paridaens *et al*, 2004) and the drug retains activity after treatment with nonsteroidal aromatase inhibitors. Exemestane is also being evaluated in the adjuvant setting. Results from direct comparison with tamoxifen are not expected for some time. However, the first data from a trial testing the sequence of tamoxifen for 2–3 years followed by exemestane for 2–3 years *vs* standard treatment with 5 years tamoxifen have recently been presented (Coombes *et al*, 2004). After a median follow-up of 30.6 months, significant reductions in first cancer-related events, disease-free survival and contralateral breast cancer were seen in the exemestane group. There was a nonsignificant increase in osteoporosis for both treatment groups, with 171 (7.4%) exemestane-treated patients and 134 (5.7%) tamoxifen-treated patients diagnosed with osteoporosis (*P*=0.05 exemestane *vs* tamoxifen). Similarly, there was a nonsignificant increase in reported fractures, with a trend towards increased fracture rate seen in the exemestane group (exemestane 3.1% *vs* tamoxifen 2.3%; *P*=0.08).

The first data from the bone subprotocol of 206 patients have recently become available (Coleman *et al*, 2004). Patients who switched from tamoxifen to exemestane lost 2.7% lumbar spine BMD after 6 months and 3.2% after 12 months. For patients who continued on tamoxifen, the mean rates of bone loss were 0.2 and 0.2% at the spine after 6 and 12 months. These differences were significant at both time points (*P*<0.0001). The rapid decline in BMD after 6 months may be due to the withdrawal of tamoxifen as much as the introduction of exemestane. Further data from measurements after 2 years will help to evaluate subsequent bone loss.

A study to investigate the effect of exemestane and letrozole on markers of bone turnover in postmenopausal women was recently updated at the San Antonio Breast Cancer Symposium (Goss *et al*, 2002). Exemestane was associated with a reduction in bone resorption markers compared with letrozole. Urinary CTX increased by 11.4% in patients treated with letrozole, but in the exemestane group it declined by 23.4%. This suggested that exemestane may have a bone-preserving action that is not seen with other aromatase inhibitors. However, the trial only involved 60 patients and follow-up was just 12 weeks. In contrast, a small substudy by Martinetti *et al* (2003) found that exemestane increased levels of bone turnover markers. A total of 23 postmenopausal patients with or without bone metastasis from metastatic breast cancer were randomised to treatment with exemestane, and samples for BAP, CTX and insulin-like growth factor were taken after 8 weeks. In the population as a whole, exemestane resulted in significant increases in both BAP and CTX (*P*<0.01).

Very recently, the preliminary results of a study comparing adjuvant exemestane with placebo in low-risk breast cancer patients were presented by Lonning *et al* (2004). At 24 months, CTX had increased from baseline by 35% with exemestane and fallen by 5% on placebo. Bone formation markers also rose, with changes in a variety of bone formation markers that were somewhat greater than might have been expected through normal bone cell coupling. The authors suggested that this reflected the anabolic action of the main metabolite of exemestane, 17-hydro exemestane. In terms of BMD, the same group reported an annual bone loss of 2.17 and 2.72% at the spine and hip, respectively (Lonning *et al*, 2004). Surprisingly, bone loss in the placebo group was greater than expected at 1.84 and 1.48% at the spine and hip, respectively, and so there was no significant difference between the two groups, although a clear trend for more rapid bone loss on exemestane was evident. None of the women were taking calcium or vitamin D supplements, and in Scandinavia at a median age of over 60 years a high proportion would be expected to be vitamin D deficient. This may have confounded the results somewhat. Overall, despite the weak androgenic effects of exemestane, it is likely that the profound suppression of circulating oestrogens caused by the potent inhibition of aromatase will over-ride this and, as with the nonsteroidal aromatase inhibitors, promote accelerated bone loss.

## TREATMENT

### Vitamin supplements

Calcium and vitamin D supplements are of benefit in preventing osteoporotic fracture in elderly women and may slow the development of osteoporosis in the over 65 years age group (Goss *et al*, 2003; Martinetti *et al*, 2003). These supplements have relatively few side effects and should be taken on a routine basis both alone and in combination with antiresorptive therapy.

### Antiresorptives

#### Bisphosphonates

The bisphosphonates are potent inhibitors of osteoclast function and can improve BMD of the lumbar spine by 5–10% in 2 years. This degree of improvement correlates with a reduction in fracture risk by approximately 50% (Eastell, 1998). Bisphosphonates of proven clinical benefit in treating osteoporosis by reducing fracture rates include alendronate (Liberman *et al*, 1995; Black *et al*, 1996, 2000; Cummings *et al*, 1998b) and risedronate (Harris *et al*, 1999; Reginster *et al*, 2000).

Presently available intravenous bisphosphonates include pamidronate, zoledronic acid and ibandronate. Zoledronic acid can be given annually at a dose of 4 mg and is as potent as daily oral preparations (Reid *et al*, 2002). In the treatment of cancer-induced bone loss, several studies have shown the benefit of using bisphosphonates to preserve BMD and presumably therefore the prevention of osteoporosis. Delmas *et al* (1997) reported on the use of risedronate to prevent bone loss associated with chemotherapy-induced early menopause.

Powles *et al* (1998) recruited more than 300 women with primary operable breast cancer from the adjuvant clodronate trial. After 2 years, patients treated with placebo lost on average 1.88% of their lumbar spine BMD. Clodronate-treated patients however lost just 0.16% (*P*=0.04). Gnant *et al* (2002) are presently investigating the effect of intravenous zoledronic acid therapy on premenopausal breast cancer patients treated with goserelin and tamoxifen or goserelin and anastrozole. Early results from this study show a significant benefit in favour of zoledronic acid for BMD of the lumbar spine and hip (*P*<0.0001) for the patients treated with zoledronic acid.

## SUMMARY

At the time of diagnosis, breast cancer patients have a somewhat higher mean BMD compared with age-matched controls. Despite this, there is evidence that patients with breast cancer are at a greater risk for developing osteoporosis. Future trends in the treatment of breast cancer are likely to advocate the use of aromatase inhibitors, which will exacerbate this problem. One way to approach this would be to screen all breast cancer patients with regular DXA scans, and treat them with bisphosphonates as appropriate. However, this would put a major strain on resources and is unlikely to be cost effective.

Consideration of risk factors for osteoporosis and fracture may help identify patients who would benefit most from investigations into their bone density. Clear evidence-based guidelines would help physicians identify which patients to investigate and provide effective treatment, and may open the door to easier access to DXA. The American Society of Clinical Oncology (ASCO) (Hillner *et al*, 2003) recognised this problem and recently published guidelines to help identify patients at high risk by a series of criteria and suggest treatment depending upon results of DXA scans of the hip or spine.

## Figures and Tables

**Table 1:** Agents causing bone loss in breast cancer patients

**Treatment**	**Study**	**Patient numbers**	**Subjects**	**Outcome**
*Chemotherapy*				
Indirect	Shapiro *et al* (2001)	49	Premenopausal women	↓ Spine BMD by 4% in 6 months
				*P*=0.0001
	Vehmanen *et al* (2001)	148	Premenopausal women	↓ Spine BMD by 7.5% in 36 months
				*P*=0.0001
Direct	Wheeler *et al* (1995)	69	Rats	↓ Bone volume, ↓ mineralising surface, ↑ osteoclast surface
				*P*<0.05
	Greep *et al* (2003)	130	Postmenopausal women	No significant change in T-score, significant changes in Z-score at lumbar spine, BMD ↓ 0.6
				*P*=0.05

*Tamoxifen*				
	Powles *et al* (1996)	125	Premenopausal women	↓ Spine BMD by 1.44% in 12 months
				*P*<0.001 (*vs* placebo)

*Ovarian ablation*				
Drug induced	Leather *et al* (1993)	19	Premenopausal women	↓ Spine BMD by 4.8% in 6 months
				*P*<0.001
Surgical	Hashimoto *et al* (1995)	244	Premenopausal women	↓ Spine BMD by 10.7% in 12 months

*Aromatase inhibitors*				
Anastrozole	Eastell *et al* (2002)	308	Postmenopausal women	↓ Spine BMD by 2.6% in 12 months
Letrozole	Goss *et al* (2003)	5187	Postmenopausal women	↑ Osteoporosis in letrozole group *vs* placebo, 5.8 *vs* 4.5%
				*P*=0.07
Exemestane	Coombes *et al* (2004)	4742	Postmenopausal women	↑ Osteoporosis in exemestane group *vs* tamoxifen group, 7.4 *vs* 5.7%
				*P*=0.05
	Lonning *et al* (2004)	147	Postmenopausal women	↓ Spine BMD at an annual rate of 2.17%

BMD=bone mineral density.

**Table 2:** Incidence of fracture in ATAC trial (adapted with permission from ATAC Trialists' Group, 2002)

	**Anastrozole (*n*=3092)**	**Tamoxifen (*n*=3094)**
Total fractures	183 (5.9%)	115 (3.7%)
Spine fractures	23 (0.7%)	10 (0.3%)
Wrist fractures	36 (1.2%)	25 (0.8%)
Hip fractures	11 (0.4%)	13 (0.4%)

ATAC trial=Arimidex, Tamoxifen Alone or in Combination trial.

**Table 3:** 6-monthly fracture rates per 100 patients at a median follow-up of 37 months in the ATAC trial (Howell, 2003; Locker and Eastell, 2003)

	**6-monthly fracture rates/100 patients**		
**Time (months)**	**Anastrozole (*n*=3092)**	**Tamoxifen (*n*=3093)**	**Anastrozole/tamoxifen 6-month hazard ratio**
6	1.11	0.99	1.14
12	0.93	0.58	1.61
18	1.36	0.69	1.98
24	1.57	0.61	2.57
30	1.39	0.96	1.45
36	1.09	0.66	1.66
42	1.50	1.37	1.09
48	1.07	0.80	1.34

ATAC trial=Arimidex, Tamoxifen Alone or in Combination trial.
